# Using AAV vectors expressing the β_2_-adrenoceptor or associated Gα proteins to modulate skeletal muscle mass and muscle fibre size

**DOI:** 10.1038/srep23042

**Published:** 2016-03-14

**Authors:** Adam Hagg, Timothy D. Colgan, Rachel E. Thomson, Hongwei Qian, Gordon S. Lynch, Paul Gregorevic

**Affiliations:** 1 Baker IDI Heart and Diabetes Institute, Melbourne, 3004, Australia; 2 Dept. of Physiology, The University of Melbourne, Melbourne, 3010, Australia; 3 Dept. of Biochemistry and Molecular Biology, Monash University, Clayton, 3800, Australia; 4 Department of Neurology, University of Washington School of Medicine, Seattle, 98195, USA

## Abstract

Anabolic β_2_-adrenoceptor (β_2_-AR) agonists have been proposed as therapeutics for treating muscle wasting but concerns regarding possible off-target effects have hampered their use. We investigated whether β_2_-AR-mediated signalling could be modulated in skeletal muscle *via* gene delivery to the target tissue, thereby avoiding the risks of β_2_-AR agonists. In mice, intramuscular administration of a recombinant adeno-associated virus-based vector (rAAV vector) expressing the β_2_-AR increased muscle mass by >20% within 4 weeks. This hypertrophic response was comparable to that of 4 weeks’ treatment with the β_2_-AR agonist formoterol, and was not ablated by mTOR inhibition. Increasing expression of inhibitory (Gαi2) and stimulatory (GαsL) G-protein subunits produced minor atrophic and hypertrophic changes in muscle mass, respectively. Furthermore, Gαi2 over-expression prevented AAV:β_2_-AR mediated hypertrophy. Introduction of the non-muscle Gαs isoform, GαsXL elicited hypertrophy comparable to that achieved by AAV:β_2_-AR. Moreover, GαsXL gene delivery was found to be capable of inducing hypertrophy in the muscles of mice lacking functional β_1_- and β_2_-ARs. These findings demonstrate that gene therapy-based interventions targeting the β_2_-AR pathway can promote skeletal muscle hypertrophy independent of ligand administration, and highlight novel methods for potentially modulating muscle mass in settings of disease.

Over 800 G-protein coupled receptor (GPCR) variants are encoded by the human genome^1^. As transmembrane receptors, the GPCRs represent the target of nearly one-third of all pharmaceuticals developed to date^2^. One of the best characterized GPCRs in skeletal muscle is the β_2_-adrenoceptor (β_2_-AR)^3^. *In vivo*, endogenous catecholamines such as adrenaline activate skeletal muscle β_2_-ARs to promote receptor interaction with stimulatory (Gαs) and inhibitory (Gαi) G-proteins^4^. The activation of these intracellular effectors differentially regulates adenylyl cyclase (AC) activity and subsequent cAMP accumulation, which impacts on several cellular mechanisms that influence the muscle phenotype^5^. Chronic stimulation of skeletal muscle β_2_-ARs through administration of β_2_-AR agonists such as clenbuterol, fenoterol and formoterol has well-characterized anabolic consequences, resulting in increased muscle mass and force-producing capacity^6^,^7^. Anabolism of skeletal muscle following β_2_-AR agonist administration has been associated with increased protein synthesis *via* stimulation of the Akt-mTOR-S6 kinase signalling axis^8^,^9^. However, β_2_-AR agonist administration can also attenuate protein degradation by repressing transcription of the muscle-specific E3 ubiquitin ligases Murf1 and Atrogin-1, and Ca^2+^-dependent proteases^10^,^11^,^12^.

Because sustained stimulation of β_2_-AR in skeletal muscle supports anabolic and anti-catabolic processes, synthetic β_2_-AR agonists have been investigated as potential therapeutics to combat the loss of muscle mass and force-producing capacity associated with conditions such as neurogenic muscle atrophy^9^,^13^,^14^, muscular dystrophy^15^,^16^,^17^, sarcopenia^6^,^18^, and cancer cachexia^7^,^19^,^20^. However, the expression of β_2_-ARs in other cell types has prompted concerns about the risks of off-target effects arising from long-term systemic administration of β_2_-AR agonists. Consequently, clinical application of these compounds for muscle wasting has remained limited. We investigated whether stimulation of β_2_-AR signalling that promotes skeletal muscle hypertrophy might be achievable by means that circumvent the potential off-target effects of β_2_-AR agonists. Specifically, we hypothesised that administering gene therapy-based interventions to alter the expression of β_2_-AR pathway components could promote skeletal muscle growth independent of β_2_-AR agonist administration. This rationale was based on the emerging development of recombinant adeno-associated virus-based vectors (rAAV vectors) as tools for therapeutic gene delivery, owing to their propensity for achieving efficacious and targeted delivery of transgenes to the skeletal muscles of mammals^21^,^22^, including humans^23^,^24^, that can sustain transgene expression for over a decade following a single treatment^24^.

Our studies identified that β_2_-AR gene delivery using rAAV vectors can promote skeletal muscle hypertrophy in mice without administration of synthetic β_2_-AR agonists. Additionally, we observed that increasing the expression of specific G-protein subunits could exert hypertrophic and atrophic effects in skeletal muscle independent of ligand administration. These studies introduce targeted gene delivery as a new strategy for manipulating the β_2_-AR signalling pathway without administering β_2_-AR agonists, to promote skeletal muscle hypertrophy.

## Results

### β_2_-AR gene delivery promotes skeletal muscle hypertrophy and protein synthesis

**Figure 1:**
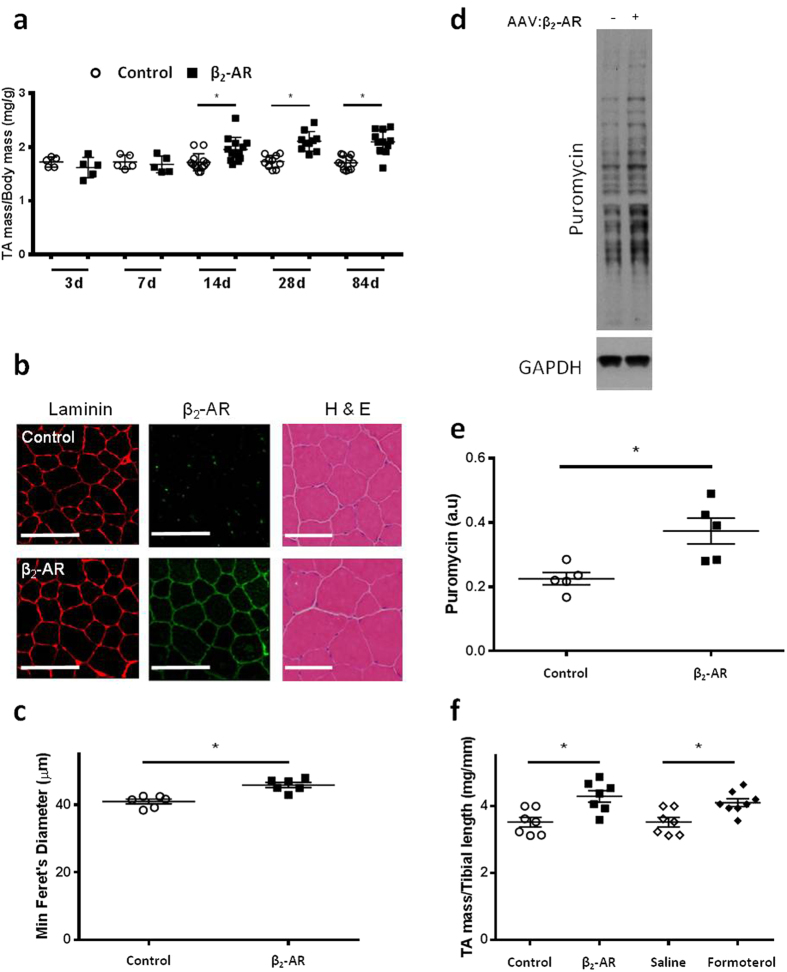
β_2_-AR gene delivery promotes skeletal muscle hypertrophy and protein synthesis. **(a)** TA muscle mass examined 3, 7, 14, 28 and 84 days after AAV:β_2_-AR administration. **(b)** Representative immunofluorescent images of β_2_-AR density (shown in green) at the myofibre membrane (shown in red) in control and AAV:β_2_-AR treated muscles four weeks post-injection (scale bar = 50 μm). Representative H&E images of TA muscle cross-sections four weeks after administration of control vector or AAV:β_2_-AR (scale bar = 50 μm). **(c)** Minimum Feret’s diameter measurements of TA muscle fibres examined four weeks after administration of control vector or AAV:β_2_-AR. **(d,e)** Representative western blots and densitometry showing puromycin incorporation in TA muscles four weeks after vector administration. **(f)** TA muscle mass four weeks after administration of control vector or AAV:β_2_-AR, or four weeks after 28 consecutive days of vehicle or formoterol administration (muscle mass is expressed relative to tibial bone length to account for differences in body mass caused by systemic effects of formoterol administration). Data are mean ± SEM. n = 3–10 mice/group. *p < 0.05.

To determine the effects of increasing β_2_-AR abundance in muscle fibres, we used adeno-associated virus-based vectors encoding the β_2_-AR (AAV:β_2_-AR) or a gene-less cassette (control) to transduce the tibialis anterior (TA) hind-limb muscles of male eight-week-old C57Bl/6 mice. Optimisation of vector doses established that injection of muscles with 1 × 10^10^ AAV:β_2_-AR vector genomes (vg) produced a 22% increase in muscle mass within 28 days of vector administration, which was maintained for at least 84 days after vector delivery (the longest time point examined) (Fig. 1a). Cross-sections of muscles immunolabelled for β_2_-AR and laminin confirmed widespread expression of β_2_-AR on the sarcolemma of transduced muscle fibres (Fig. 1b), and an increase in the diameter of muscle fibres in treated muscles (Fig. 1c). Muscles administered AAV:β_2_-AR exhibited increased rates of protein synthesis as measured by acute puromycin incorporation^25^ (Fig. 1d,e). To assess whether AAV:β_2_-AR administration altered the muscle fibre type distribution, sections of treated TA muscles were examined *via* histochemical reaction to estimate succinate dehydrogenase (SDH) activity and immunolabelled for prevalence of the myosin type IIa isoform. Muscles examined four weeks after administration of AAV:β_2_-AR or control vector did not exhibit a difference in the proportion of fibres expressing the type IIa myosin heavy chain isoform, or the activity of SDH (Supplementary Fig. S1).

To determine if the magnitude of hypertrophy induced *via* administration of AAV:β_2_-AR was comparable to that achieved by treating muscles with anabolic β_2_-AR agonists, additional cohorts of mice were administered AAV:β_2_-AR, or daily injections of formoterol (100 μg/kg) for 28 days. We observed that a single administration of AAV:β_2_-AR and 28 consecutive days of formoterol administration produced comparable increases in muscle mass (Fig. 1f, normalised relative to tibial bone length rather than body mass to account for the effect of changes in lean mass in mice receiving formoterol).

### Muscle hypertrophy induced by β_2_-AR gene delivery is not inhibited by rapamycin

**Figure 2:**
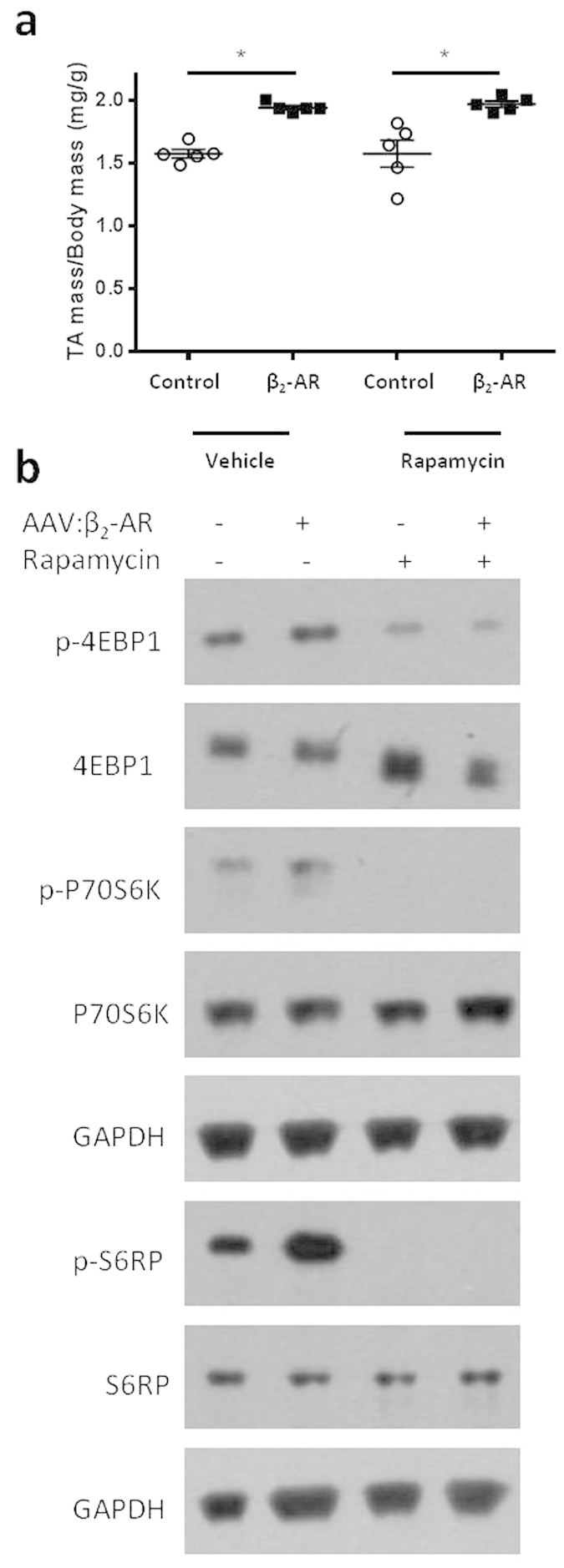
Muscle hypertrophy induced by β_2_-AR gene delivery is not inhibited by Rapamycin. **(a)** TA muscle mass four weeks post control vector or AAV:β_2_-AR injection and daily administration of vehicle or rapamycin. **(b)** Representative western blots indicating phosphorylated and total levels of 4EBP1, P70S6K, S6RP and GAPDH as a loading control. (n = 5 mice/group) Data are mean ± SEM. *p < 0.05.

As repeated administration of anabolic β_2_-AR agonists has been reported to promote skeletal muscle growth *via* signalling dependent on the activation of mTOR, we investigated whether hypertrophy as a consequence of AAV:β_2_-AR administration was also associated with mTOR-driven processes. Western blot analysis of TA muscles examined 14 days after administration of AAV:β_2_-AR or control vector revealed a significant increase in phosphorylation of S6RP but not the upstream regulators Akt and mTOR (Supplementary Fig. S2). Additional mice administered AAV:β_2_-AR were treated with 28 daily injections of rapamycin, an inhibitor of mTOR, to further test whether mTOR activity is necessary to achieve muscle hypertrophy associated with increased β_2_-AR expression. Increases in muscle mass and myofibre diameter as a consequence of transducing muscles with AAV:β_2_-AR did not differ between animals receiving rapamycin or vehicle for 28 days (Fig. 2a and Supplementary Fig. S3). Furthermore, whereas administration of AAV:β_2_-AR increased phosphorylation of P70S6K and S6RP, rapamycin administration inhibited phosphorylation of these two proteins and 4EBP1 (Fig. 2b and Supplementary Fig. S3), thereby confirming the bioactivity of the rapamycin regimen used.

### GαsL and Gαi2 gene delivery have opposing effects on TA muscle mass

**Figure 3:**
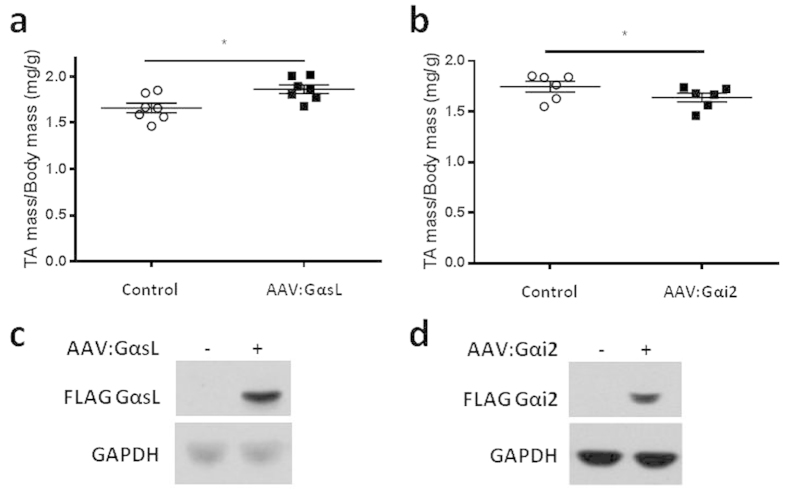
GαsL and Gαi2 gene delivery have opposing effects on TA muscle mass. **(a)** TA muscle mass four weeks after administration of control vector or AAV:GαsL. **(b)** TA muscle mass four weeks after administration of control vector or AAV:Gαi2. **(c)** Western blot analysis of FLAG-tagged GαsL in TA muscles injected with AAV:GαsL. **(d)** Western blot analysis of FLAG-tagged Gαi2 in TA muscles injected with AAV:Gαi2. Data are mean ± SEM. n = 6–7 mice/group. *p < 0.05.

**Figure 4:**
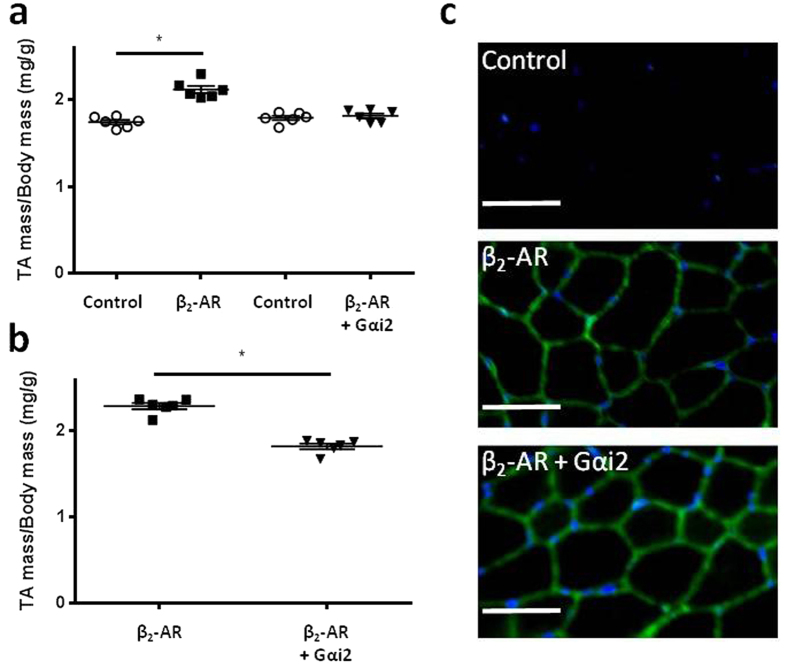
Gαi2 gene delivery inhibits the anabolic effects of β_2_-AR gene delivery. **(a)** TA muscle mass four weeks after administration of control vector, AAV:β_2_-AR or AAV:β_2_-AR and AAV:Gαi2. **(b)** TA muscle mass four weeks after administration of AAV:β_2_-AR with control vector or AAV:β_2_-AR with AAV:Gαi2. **(c)** Representative immunofluorescent images confirming β_2_-AR over-expression (shown in green) and nuclei (shown in blue) four weeks after vector administration (scale bar = 50 μm). Data are mean ± SEM. n = 6 mice/group. *p < 0.05.

As β_2_-adrenoceptors utilise stimulatory (Gαs) and inhibitory (Gαi) G-proteins to propagate intracellular signalling, we investigated whether treating the TA muscles of mice with AAV vectors that increase expression of either GαsL or Gαi2 affected muscle mass. Injection of TA muscles with AAV:GαsL four weeks prior to examination increased mass by 8% (Fig. 3a), whereas administration of AAV:Gαi2 was associated with a 6% decrease in muscle mass (Fig. 3b). Expression of FLAG-tagged GαsL and Gαi2 proteins was confirmed by Western blot (Fig. 3c,d respectively). As our data showed Gαi2 to be a negative regulator of muscle mass, we investigated whether increased expression of Gαi2 could attenuate the anabolic effects of β_2_-AR gene delivery. Cohorts of mice received intramuscular injections of AAV:β_2_-AR in combination with AAV:Gαi2 or control vector. Consistent with effects reported in Fig. 1, mice administered AAV:β_2_-AR and control vector demonstrated TA muscle hypertrophy (Fig. 4a). However, co-administration of AAV:Gαi2 completely prevented the anabolic effects of AAV:β_2_-AR administration (Fig. 4a). To validate this observation, additional mice received bilateral TA muscle injections of AAV:β_2_-AR in combination with AAV:Gαi2 or control vector. Four weeks after vector administration, TA muscles administered AAV:β_2_-AR with control vector exhibited a 20% increased mass compared with contralateral muscles co-administered AAV:β_2_-AR with AAV:Gαi2 (Fig. 4b). Immunolabelling of muscles confirmed comparable expression of β_2_-AR between treatment conditions (Fig. 4c).

### GαsXL gene delivery promotes muscle hypertrophy independent of β_1_- and β_2_- adrenoceptors

**Figure 5:**
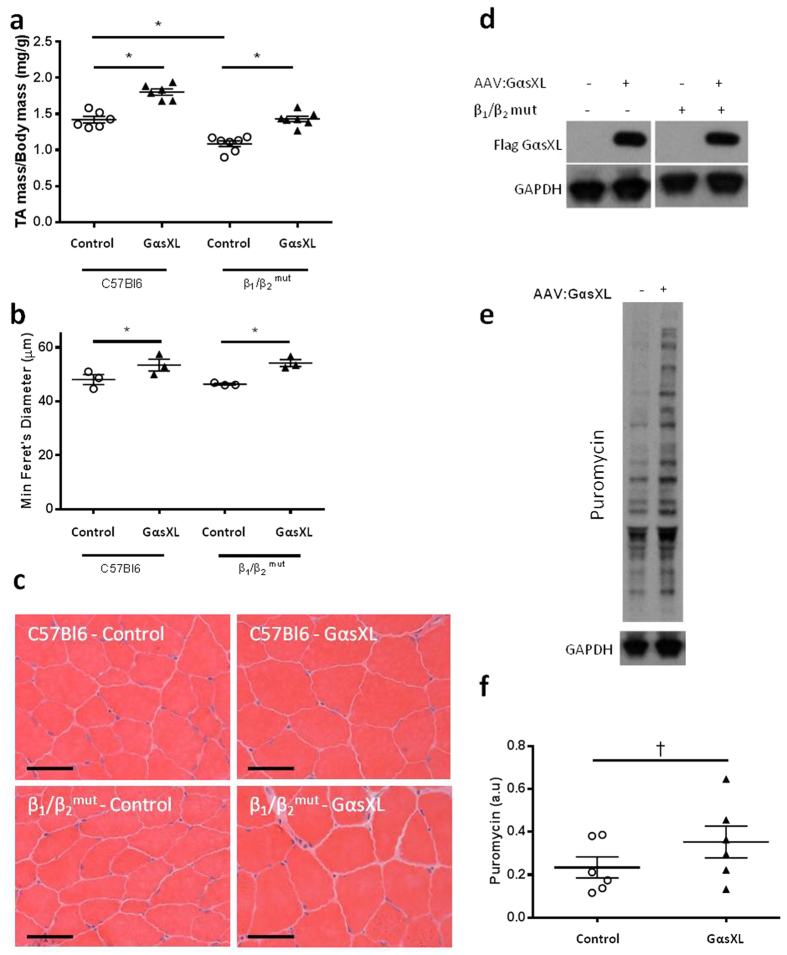
GαsXL gene delivery promotes muscle hypertrophy independent of β_1_- and β_2_- adrenoceptors. **(a)** TA muscle mass four weeks after administration of control vector or AAV:GαsXL to C57Bl6 or β_1_/β_2_^mut^ mice. **(b)** Minimum Feret’s diameter measurements of TA muscle fibres from C57Bl6 and β_1_/β_2_^mut^ mice treated with control vector or AAV:GαsXL. **(c)** Representative H&E images of TA muscle cross-sections examined four weeks after vector administration (scale bar = 50 μm). **(d)** Western blot confirming the presence of flag tagged GαsXL in treated muscles. **(e,f)** Western blot and densitometry displaying puromycin incorporation in TA muscles four weeks post treatment. Data points are portrayed together with mean ± SEM. n = 3–6 mice/group. *p < 0.05. ^†^p = 0.05.

The extra-large isoform of Gαs, GαsXL, is predominantly expressed in neurons and has been reported to promote increased cAMP activity compared to GαsL in a cell culture model of β_2_-AR activation^26^,^27^. Reasoning that ectopic expression of GαsXL in skeletal muscle could confer greater effects on muscle mass than those achieved via GαsL gene delivery, we examined the effects of administering AAV:GαsXL to the TA muscles of C57Bl6 mice. Muscles examined 28 days after administration of AAV:GαsXL exhibited a 27% increase in mass (Fig. 5a) and a significant increase in myofibre diameter (Fig. 5b) compared to contralateral muscles receiving control vector. Administration of AAV:GαsXL was also associated with an increased proportion of muscle fibres expressing the type IIa myosin heavy chain isoform, although no accompanying significant change in SDH activity was observed (Supplementary Fig. S4). Consistent with the stimulatory effects of AAV:β_2_-AR administration upon protein synthesis (reported in Fig. 1d), the muscles of wild-type mice treated with AAV:GαsXL also demonstrated markedly increased rates of protein synthesis, as estimated from puromycin incorporation (Fig. 5e,f). To determine whether muscle hypertrophy associated with AAV:GαsXL administration was dependent on β-AR activity, we administered AAV:GαsXL to mice lacking functional β_1_- and β_2_-ARs (β_1_/β_2_^mut^ mice)^28^,^29^. Four weeks after administration of AAV:GαsXL to β_1_/β_2_^mut^ mice, treated TA muscles exhibited a 35% increased mass (Fig. 5a), and significantly increased muscle fibre diameter (Fig. 5b,c), compared with contralateral muscles administered control vector. Comparable expression of GαsXL was confirmed in the treated muscles of C57Bl6 and β_1_/β_2_^mut^ mice by western blot probing for flag-tagged GαsXL (Fig. 5d).

## Discussion

Although synthetic β_2_-AR agonists exert anabolic and anti-catabolic effects on mammalian skeletal muscles, their clinical application for muscle wasting has been limited by concerns regarding potential off-target effects^30^,^31^,^32^. Our findings demonstrate a novel method of stimulating β_2_-AR signalling in muscle fibres, based on the use of recombinant AAV vectors to deliver β_2_-AR or Gαs expression constructs. As recombinant viral vectors can be configured to achieve tissue-specific transgene delivery and expression by combining the cell-selective tropism of vectors with cell-specific transcription/translation control elements^33^,^34^, muscle-directed gene delivery may hold potential as a strategy for manipulating the β_2_-adrenergic network without the need to repeatedly administer potent β_2_-AR agonists. The benefits of such an approach could provide the means to effectively promote anabolic signalling in the target tissue (i.e. skeletal muscle), while minimising the potential for incurring off-target effects in other tissues.

Using rAAV vectors to enhance β_2_-AR expression in mouse limb muscles promoted increases in myofibre size and augmented protein synthesis. The hypertrophic effects of AAV:β_2_-AR administration were comparable in magnitude to those achieved with repeated administration of the potent β_2_-AR agonist formoterol. We did not find evidence that activation of mTOR was required to support muscle hypertrophy induced by β_2_-AR gene delivery, which contrasts with reports of β_2_-AR agonist-induced skeletal muscle hypertrophy requiring mTOR signalling^9^. As myogenic cells can elicit an anabolic response downstream of the β_2_-AR *via* the PKC/GSK3β signalling axis^35^, hypertrophy as a result of AAV:β_2_-AR administration could utilise similar mechanisms. These observations point to other possible advantages of developing skeletal-muscle-directed gene delivery as an alternative method for manipulating β_2_-adrenergic signalling *in vivo*. Further research is warranted to more comprehensively examine the similarities and differences between drug- and gene-based interventions targeting this signalling system in striated muscle.

Having established that β_2_-AR gene delivery can stimulate skeletal muscle hypertrophy, we investigated whether β_2_-AR-mediated effects could be potentiated by increasing the abundance of specific Gα protein subunits operating as signalling substrates for the β_2_-AR. Increasing expression of GαsL promoted a modest hypertrophic effect compared with AAV:β_2_-AR administration. Broadly, this stimulatory role of Gαs is consistent with earlier work demonstrating that muscle mass is reduced in Gαs knockout mice^36^. In contrast, increasing expression of Gαi2 alone produced muscle atrophy, and more significantly, co-delivery of AAV:Gαi2 with AAV:β_2_-AR completely prevented the hypertrophic effects of β_2_-AR gene delivery. These findings are consistent with the possibility that Gαi2 possesses comparatively greater (relative to Gαs) affinity for interaction with the β_2_-AR, or that increased abundance of Gαi2 can outcompete Gαs for interaction with the β_2_-AR.

Although Gαi2 is considered to operate in opposition to Gαs, the inhibitory effects of over-expressing wild-type Gαi2 in muscle fibres described herein contrast with studies that documented protein accretion and cell growth after transducing myogenic cells with a constitutively active Gαi2^Q205L^ mutant^35^. Global embryonic knock-out of Gαi2 produces mice with muscles of reduced myofibre size, although the animals also suffer from a lethal intestinal phenotype and immune cell defects that likely compromise interpretation of the muscle attributes^37^,^38^. Stronger evidence from cell culture studies supports a role for Gαi2 in guiding the proliferation and differentiation of myogenic progenitors^35^,^37^. While the mechanisms by which Gαi2^Q205L^ promotes cell proliferation and recruitment lie outside the scope of the present study, the differences in effects of Gαi2^Q205L^ reported elsewhere versus the effects of wild-type Gαi2 reported here appear to be attributed at least in part to differential actions within myogenic progenitor cells versus muscle fibres. Additionally, it cannot yet be ruled out that the Gαi2^Q205L^ mutant isoform does not exert different effects on downstream signalling targets or is affected differently by feedback mechanisms.

Although the GNAS gene encodes for the GαsL G-protein in skeletal muscle, alternate Gαs transcript variants are encoded by GNAS in other cell types. As the predominantly neuroendocrine GαsXL variant^39^ has been reported to stimulate increased cAMP activity when compared with GαsL^26^,^27^, we reasoned that expressing GαsXL in muscle fibres may cause a hypertrophic response in skeletal muscle. Supporting this hypothesis, we found that muscles treated with AAV:GαsXL demonstrated a significant hypertrophic response with effects comparable to those achieved by treating muscles with either AAV:β_2_-AR or formoterol. Muscles treated with AAV:GαsXL exhibited an increased proportion of myofibres expressing the type IIa myosin heavy chain isoform, whereas no such effect was noted in muscles receiving AAV:β_2_-AR. These findings lend support to the idea that the two vector-based interventions have differing effects upon the physiological properties of treated skeletal muscles. The marked muscle hypertrophy with administration of AAV:GαsXL was recapitulated in mice lacking functional β_1_- and β_2_-ARs, which do not exhibit an anabolic response when administered anabolic β-agonists^40^. These results demonstrate that expression of GαsXL in skeletal musculature confers anabolic adaptations that are not dependent on active β_1_- and β_2_-ARs. It is not clear whether the observed muscle hypertrophy is a product of GαsXL possessing constitutive activity, or whether GαsXL proteins may be activated by other GPCRs in muscle fibres. Other receptors in muscle that could function as activators of ectopically expressed GαsXL include Fzd7 (previously implicated in regulation of myogenic cells^41^) and PTH1 (which promotes GαsXL activation in other tissues^42^,^43^). Collectively, these findings highlight fascinating aspects of how G proteins can modulate muscle attributes, and support the rationale for further study.

In summary, this study presents the first demonstration that treatment of mammalian skeletal muscle fibres with recombinant AAV vectors expressing the β_2_-AR or GαsXL promote changes in protein turnover that favour myofibre hypertrophy. These proof-of-concept studies focused on manipulating β-adrenergic signalling in individual limb muscles, and demonstrate the feasibility of stimulating anabolic signalling *via* the β_2_-AR signalling pathway without administering β_2_-AR agonists. The findings provide important insight into GPCR signalling in skeletal muscle, with implications for developing novel interventions for muscle wasting conditions. Given the uncertainties regarding the long-term administration of potent β_2_-AR agonists to patients, developing new strategies by which to promote anabolic β_2_-AR signalling in skeletal muscle without using β_2_-AR agonists warrants deeper investigation. This includes systemic administration of AAV vectors to achieve body-wide transduction of skeletal muscles. The findings reported here provide valuable insight into a new intervention concept, upon which such studies could be developed. Comprehensively investigating the consequences of muscle-directed gene delivery in mouse models of muscle wasting will help to determine the therapeutic potential of this novel strategy, including effects on muscle functionality and other organ systems.

## Methods and Materials

All reagents were purchased from Sigma-Aldrich unless otherwise stated.

### Animal Experiments

*In vivo* procedures were conducted in accordance with the relevant codes of practice for the care and use of animals for scientific purposes (National Institute of Health, 1985, and the National Health & Medical Research Council of Australia 2013). All experimental protocols were approved by the Alfred Medical and Education Precinct Animal Ethics Committee (AMREP AEC). All surgical procedures were performed under inhalation of isoflurane in medical oxygen with post-operative analgesia. Eight to 10 week old, male, C57Bl/6 and β_1_/β_2_ mutant (β_1_/β_2_^mut^) mice were used for all experiments. Animals were fed standard chow diets with access to drinking water *ad libitum* while housed under a 12-hour light dark cycle. β_1_/β_2_^mut^ mice were sourced and bred as described previously^28^. Doses of AAV:β_2_-AR, AAV:Gαi2, AAV:GαsL (1 × 10^10^ vg) and AAV:GαsXL (1 × 10^9^ vg) vectors (identified from preliminary dose-optimisation experiments) were diluted in 30 μl of Hank’s buffered saline solution (HBSS) and directly injected into the TA muscle. Control injections consisted of the administration of a viral vector lacking a functional gene into the contralateral limb. For systemic β-agonist treatments, intraperitoneal injections of formoterol at 100 μg/kg or saline were administered daily for 28 days. Rapamycin (ApexBio) was dissolved in DMSO to a stock concentration of 10 mg/ml, and a working concentration of rapamycin was formulated in a solution of 0.1% carboxymethylcellulose and 0.125% polysorbate-80. Mice received 2 mg/kg of rapamycin one day before and at the time of AAV:β_2_-AR administration by intraperitoneal injection. Mice were treated daily until experimental endpoint. For puromycin administration, mice received 0.04 μmol/g of puromycin (Life Technologies) via intraperitoneal injection exactly 30 min before experimental endpoint. Experimental endpoints were 28 days post viral vector administration unless indicated otherwise. Mice were humanely killed *via* cervical dislocation and the muscles rapidly excised and weighed before subsequent processing.

### Antibodies

All antibodies were purchased from Cell Signaling Technologies and used at a dilution of 1:1000, except anti-puromycin and anti-laminin B2 (Millipore) which were used at 1:5000 and 1:250 respectively, anti-β_2_-AR (MBL) 1:500 and anti-GAPDH (Santa Cruz Biotechnology) 1:10000.

### Recombinant AAV vector design and production

Traditional cloning techniques were used to generate cDNA constructs encoding Adrb2 (β_2_-AR), Gnai2 (Gαi2), GnasL (GαsL) and GnasXL (GαsXL) (synthesized by GenScript) which were cloned into an AAV expression plasmid consisting of a cytomegalovirus (CMV) promoter and SV40 poly-A region flanked by AAV2 terminal repeats. The Gαi2, GαsL and GαsXL cDNA construct also included a flag-tag coding region at the 5′ end of the coding sequences. Viral vector production was performed as described previously^21^. Briefly, HEK-293 cells were plated at a density of 3.2–3.8 × 10^6^ cells on a 10 cm culture dish, 8–16 hours before transfection with 10 μg of a vector genome-containing plasmid and 20 μg of the packaging/helper plasmid pDGM6 by calcium phosphate precipitation. At 72 hours post transfection, the medium and cells were collected and homogenized through a microfluidizer (Microfluidics) before 0.22 μm clarification (Millipore). Purification of viral particles from crude lysates was performed using affinity chromatography over a heparin affinity column (HiTrap, Amersham), and ultracentrifugation overnight prior to re-suspension in sterile physiological Ringer’s solution. The purified vector preparations were titered with a customized sequence-specific quantitative PCR-based reaction (Life Technologies).

### Western blotting

Muscles were homogenized in NP-40 lysis buffer containing protease and phosphatase inhibitor cocktails. Lysates were centrifuged at 15,000 g for 20 min at 4 °C, protein concentration was determined using a BCA protein assay kit (Thermo Scientific) and samples denatured for 5 min at 95 °C. Protein fractions were resolved by SDS-PAGE using pre-cast 4–12% Bis-Tris gels (Life Technologies), blotted onto nitrocellulose membranes (BioRad) and incubated with the appropriate primary antibody and detected as described previously^44^. Quantification of labelled western blots was performed using ImageJ pixel analysis (NIH Image software), and data normalized to corresponding GAPDH controls.

### Histological analysis

Harvested muscles were embedded in optimum cutting temperature (OCT) cryoprotectant (Sakura Finetek) and frozen in liquid nitrogen-cooled isopentane. Frozen samples were cryosectioned at 10 μm thickness using a Leica CM1950 cryostat. Cross-sections were fixed in room temperature methanol and stained with hematoxylin and eosin as described previously^44^. Stained sections of muscles were examined using a light microscope with digital camera (BX-50, Olympus), to capture images analysed for muscle fibre morphology. Minimum Feret’s diameter of myofibres was quantified using ImageJ software analysis. Up to eight fields of view were captured from the same locations within each TA muscle and contrast adjusted to gate fibres based on numerical threshold, >200 myofibres were measured per muscle. Histochemical estimation of SDH activity and immunolabelling of the type-IIa myosin heavy chain isoform was performed on 10 μm thick cryosections of harvested TA muscles as previously described^45^. Images were captured (Axio Imager D1 microscope, Carl Zeiss). SDH activity was estimated by capturing four 100x magnification brightfield images per TA muscle, and quantifying pixel density for each muscle fibre within the identified fields *via* ImageJ software analysis. Myosin type IIa positive fibres were counted and expressed relative to total number of myofibres counted per section (>600 fibres samples per muscle).

### Immunofluorescence Microscopy

OCT-frozen TA samples were cryosectioned at 8 μm thickness, fixed in methanol, washed in potassium phosphate buffered saline (KPBS) containing gelatin and blocked in a solution consisting of Tween-20, BSA, gelatin and KPBS. The sections were incubated in anti-laminin B2 and anti-β_2_-AR primary antibodies overnight at 4 °C. Alexa-Fluor-488 and -594 secondary goat antibodies (Life Technologies) were used to detect β_2_-AR and laminin B2 primary antibodies respectively, followed by 3 min incubation in DAPI nuclear stain (Life Technologies) and mounting in HardSet Vectashield (Vector Laboratories). Images were captured using a BX61 light microscope (Olympus).

### Statistical Analysis

All data are represented as the mean ± SEM. A paired Student’s t-test was used for comparisons between two conditions. Two-way analysis of variance (ANOVA) was used to measure statistical differences between multiple conditions with Tukey’s *post hoc* analysis for specific group comparisons. All significant differences are reported with p < 0.05.

## Additional Information

**How to cite this article**: Hagg, A. *et al.* Using AAV vectors expressing the β_2_-adrenoceptor or associated Gα proteins to modulate skeletal muscle mass and muscle fibre size. *Sci. Rep.* **6**, 23042; doi: 10.1038/srep23042 (2016).

## Supplementary Material

Supplementary Information
