# Supplementary material for: Using AAV vectors expressing the β2-adrenoceptor or associated Gα proteins to modulate skeletal muscle mass and muscle fibre size
Source: Sci Rep. 2016 Mar 14;6:23042. doi: 10.1038/srep23042 (PMC4789796; doi:10.1038/srep23042)
Supplement: Supplementary Information [file srep23042-s1.pdf]

Scientific Reports: SREP-15-27656

Using AAV vectors expressing the β2-adrenoceptor or associated Gα proteins to modulate skeletal muscle mass and muscle fibre size

Adam Hagg[1,2], Timothy D. Colgan[1,2], Rachel E. Thomson[1], Hongwei Qian[1], Gordon S. Lynch[2], Paul Gregorevic[1,2,3,4]

Supplementary Figures 1, 2, 3 & 4

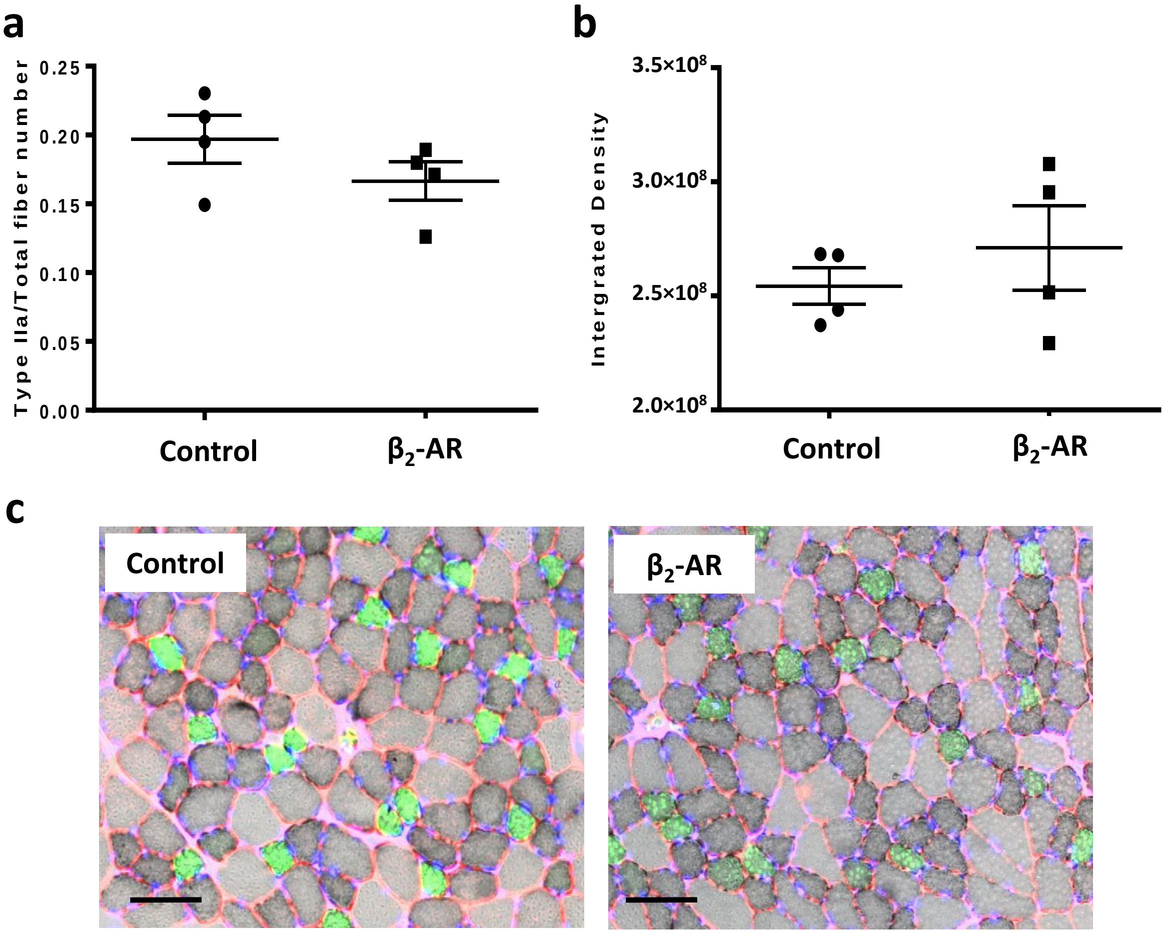

Supp Fig S1. β_2-AR gene delivery does not alter type IIa myosin expression or succinate dehydrogenase activity. (a) Proportion of muscle fibres positive for type IIa myosin heavy chain at four weeks after administration of AAV:β_2-AR or control vector. **(b)** Integrated pixel density of TA muscle cryosections histochemically reacted for SDH activity four weeks after administration of AAV:β_2-AR or control vector. **(c)** Merged images showing myofibre membrane (red), nuclei (blue), type IIa myosin isoform (green) and SDH staining (grey). Data are mean ± SEM. Scale bar = 100 μm. n = 4 mice/group.

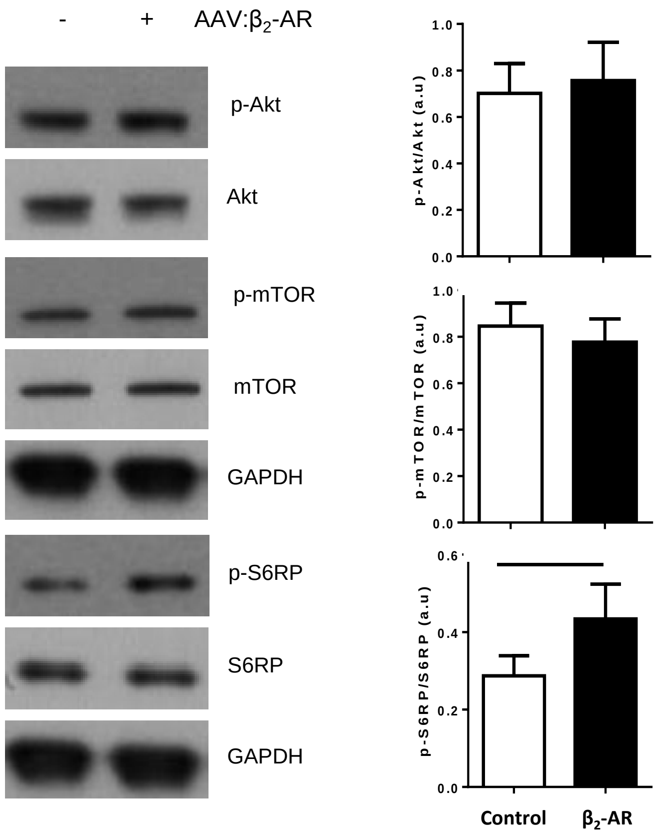

Supp Fig S2. Muscle hypertrophy induced by β_2-AR gene delivery does not increase Akt/mTOR phosphorylation. (a) Representative western blot analysis of TA muscles for abundance of phosphorylated and total of Akt, mTOR, S6RP, and GAPDH as a loading control. Data are mean ± SEM. n = 5 mice/group, *p <0.05.

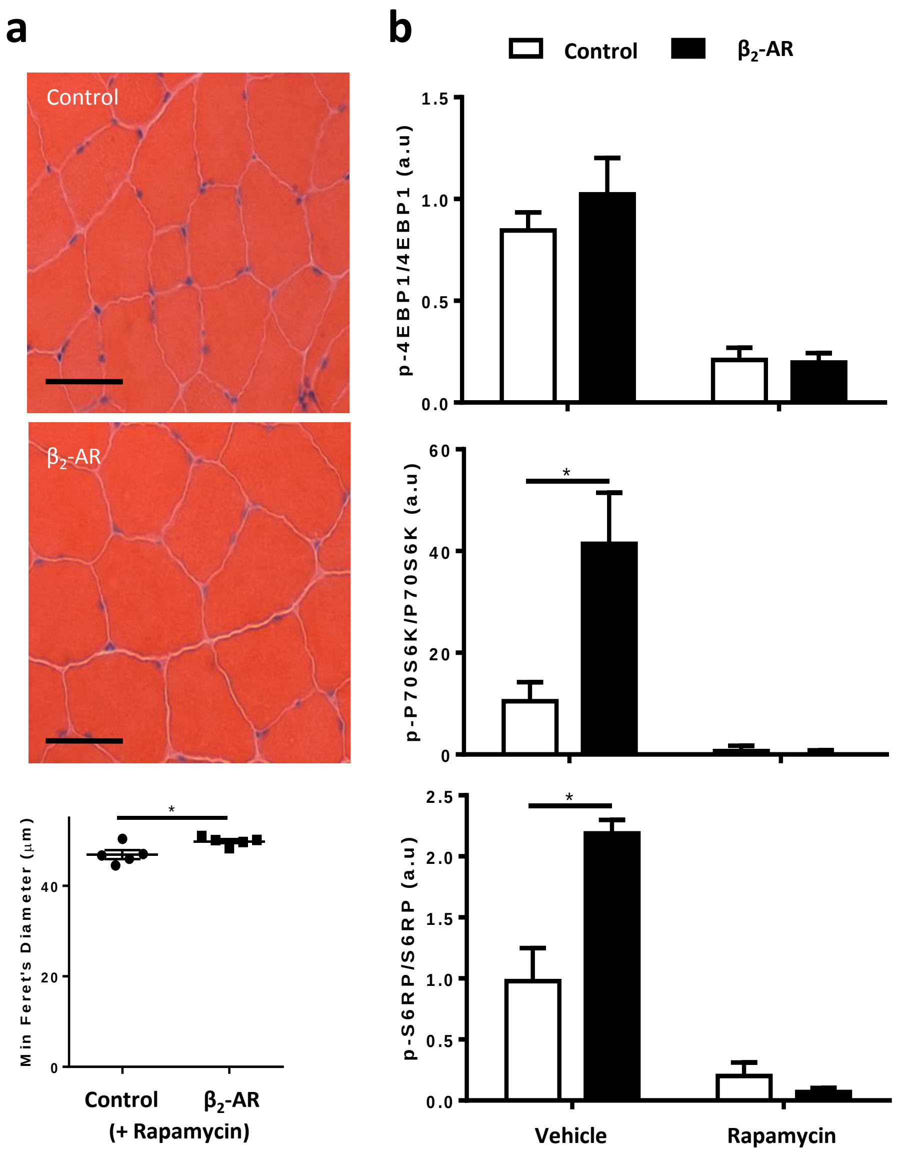

Supp Fig S3. Rapamycin does not block the hypertrophic effects of AAV:β_2-AR. **(a)** Representative H&E images (scale bar = 50 μm) and minimum Feret's diameter measurements of muscle fibres from TA muscles examined four weeks after administration of control vector or AAV:β_2-AR accompanied by daily injections of vehicle or rapamycin. **(b)** Densitometric analysis of representative western blots (featured in Fig 2b) of TA muscle lysates examined four weeks after administration of control vector or AAV:β_2-AR and daily administration of vehicle or rapamycin. Data are mean ± SEM. n = 5 mice/group. *$p \leq 0.05$.

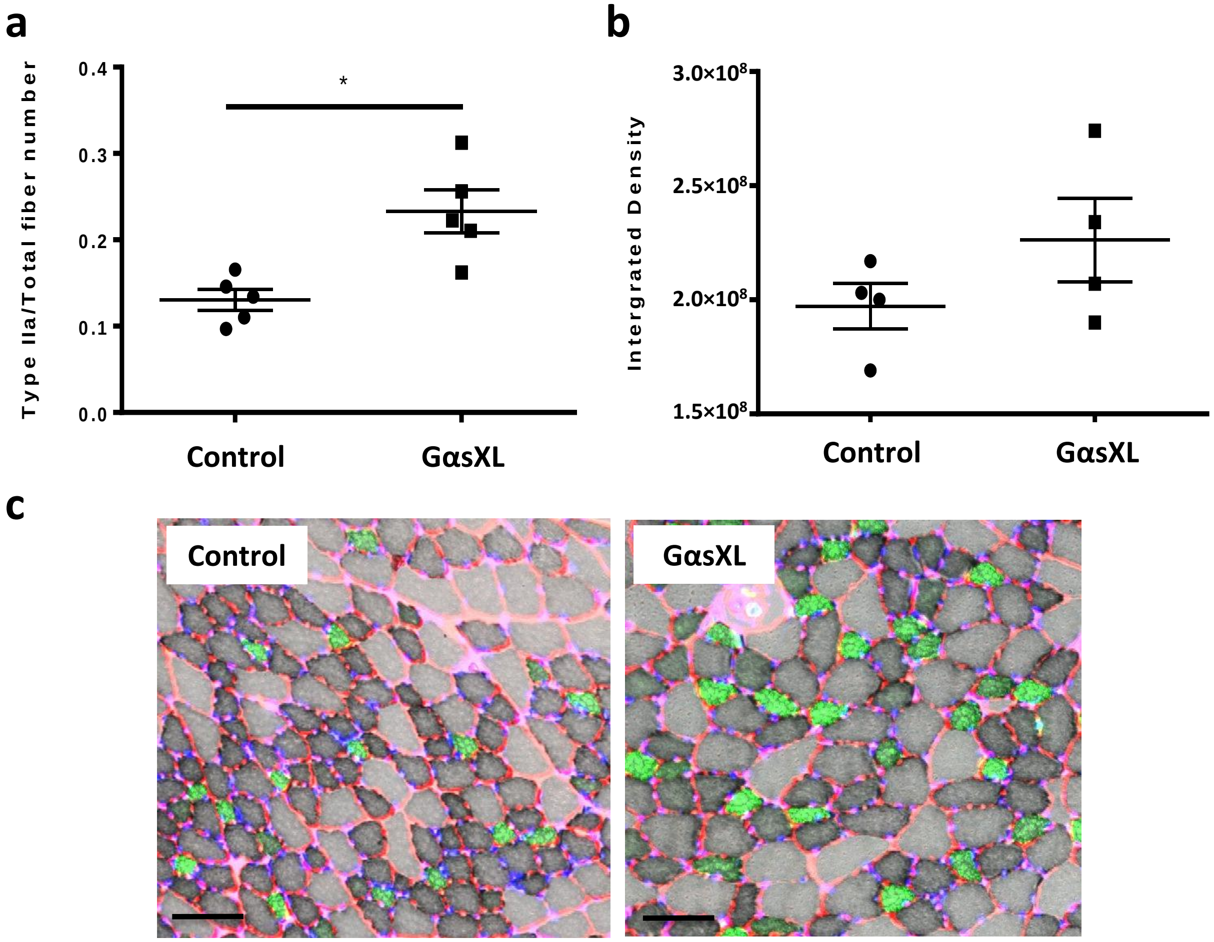

Supp Fig S4. AAV:GαsXL increases the proportion of muscle fibres expressing type IIa myosin heavy chain. (a) Proportion of myosin type IIa-positive muscle fibres four weeks after injection of muscles with control vector or AAV:GαsXL. **(b)** Integrated pixel density of TA muscle cryosections histochemically reacted for SDH activity four weeks after administration of AAV:GαsXL or control vector. **(c)** Representative merged images showing muscle fibre membrane (red), nuclei (blue), type IIa myosin isoform (green) and SDH activity (grey). Scale bar = 100 μm. Data are mean ± SEM. n = 4 mice/group. *p ≤0.05.
